# Wave 2 of the Multilingual Eye-Movement Corpus (MECO): New text reading data across languages

**DOI:** 10.1038/s41597-025-05453-3

**Published:** 2025-07-10

**Authors:** Noam Siegelman, Sascha Schroeder, Yaqian Borogjoon Bao, Cengiz Acartürk, Niket Agrawal, Lena S. Bolliger, Jan Brasser, César Campos-Rojas, Denis Drieghe, Dušica Filipović Đurđević, Sofya Goldina, Romualdo Ibáñez Orellana, Lena A. Jäger, Ómar I. Jóhannesson, Anurag Khare, Nik Kharlamov, Hanne B. S. Knudsen, Árni Kristjánsson, Charlotte E. Lee, Jun Ren Lee, Marina P. T. Leite, Simona Mancini, Nataša Mihajlović, Ksenija Mišić, Miloslava Orekhova, Olga Parshina, Milica Popović Stijačić, Athanassios Protopapas, David R. Reich, Anurag Rimzhim, Rui Rothe-Neves, Thais M. M. Sá, Andrea Santana-Covarrubias, Irina Sekerina, Heida M. Sigurdardottir, Anna Smirnova, Priyanka Srivastava, Elisangela N. Teixeira, Ivana Ugrinic, Kerem Alp Usal, Karolina Vakulya, Ark Verma, João M. M. Vieira, Denise H. Wu, Jin Xue, Sunčica Zdravković, Junjing Zhuo, Laoura Ziaka, Victor Kuperman

**Affiliations:** 1 Hebrew University of Jerusalem, Jerusalem, Israel; 2 University of Goettingen, Goettingen, Germany; 3 McMaster University, Hamilton, ON Canada; 4 Jagiellonian University, Kraków, Poland; 5 Indian Institute of Technology Kanpur, Kanpur, India; 6 University of Zurich, Zürich, Switzerland; 7 Pontificia Universidad Católica de Valparaíso, Valparaíso, Chile; 8 Millennium Nucleus for the Science of Learning (MiNSoL), Talca, Chile; 9 University of Southampton, Southampton, UK; 10 University of Belgrade, Belgrade, Serbia; 11 University of Novi Sad, Novi Sad, Serbia; 12 CNRS Aix-Marseille Université, Aix-en-Provence, France; 13 HSE University, Moscow, Russia; 14 University of Potsdam, Potsdam, Germany; 15 University of Iceland, Reykjavik, Iceland; 16 Aalborg University, Aalborg, Denmark; 17 National Taiwan Normal University, Taipei, Taiwan; 18 Universidade Federal de Minas Gerais, Belo Horizonte, Brazil; 19 Basque Center on Cognition Brain and Language, Donostia-San Sebastian, Spain; 20 Ikerbasque, Bilbao, Spain; 21 Singidunum University, Belgrade, Serbia; 22 Middlebury College, Middlebury, VT USA; 23 University of Oslo, Oslo, Norway; 24 College of the Holy Cross, Worcester, MA USA; 25 Universidade Federal de Lavras, Lavras, Brazil; 26 College of Staten Island of the City University of New York, Staten Island, NY USA; 27 University of Groningen, Groningen, Netherlands; 28 International Institute of Information Technology Hyderabad, Hyderabad, India; 29 Universidade Federal do Ceará, Fortaleza, Brazil; 30 Middle East Technical University, Ankara, Türkiye; 31 University of Plymouth, Plymouth, England; 32 National Central University, Taoyuan City, Taiwan; 33 Beijing Institute of Technology, Beijing, China; 34 Northeast Normal University, Changchun, China; 35 Oslo University Hospital, Oslo, Norway

**Keywords:** Human behaviour, Databases

## Abstract

This paper reports the Wave 2 expansion of the Multilingual Eye-Movement Corpus (MECO), a collaborative multi-lab project collecting eye-tracking data on text reading in a variety of languages. The present expansion comes with new eye-tracking data of N = 654 from 13 languages, collected in 16 labs over 15 countries, including in several languages that have little to no representation in current eye-tracking studies on reading. MECO also contains demographic, language use, and other individual differences data. This paper makes available the first-language reading data of MECO Wave 2 and incorporates reliability estimates of all tests at the participant and item level, as well as other methods of data validation. It also reports the descriptive statistics on all languages, including comparisons with prior similar data, and outlines directions for potential reuse.

## Background & Summary

A central goal of language research is to develop theoretical and computational accounts of linguistic structure, function, and behaviour that can generalize over the astounding diversity of world languages. However, research into language acquisition and use demonstrably suffers from a bias towards and an over-reliance on data from speakers of Indo-European languages in general and English in particular^1,2^. Among the sub-fields of cognitive sciences where such bias is salient is the study of reading^3,4^. Our focus is on reading research that uses eye-tracking as the experimental paradigm, since eye movements have been shown to be reliable and valid indices of real-time cognitive processes unfolding during reading^5^. For instance, Siegelman *et al*.^6^ bibliometric analysis of eye-tracking studies of reading between 2000 and 2018 revealed that this literature only addresses 28 unique languages out of roughly 4,000 written languages that exist in the world (https://www.ethnologue.com/). Over 50% of those papers have the English language as their subject, and there is a strong overall bias towards alphabetic and Indo-European languages (see also^7^). There is an even greater deficit of studies dedicated to direct cross-linguistic comparisons of two or more languages. Thus, the progress of research into reading is undermined by paucity of high-quality data that represent multiple languages and uses comparable text stimuli, participants, and equipment. Such data can enable researchers to move beyond the idiosyncrasies of a specific language or population both in their theorizing and computational modelling (notably, leading models of eye movement control in reading are based on either Chinese, English, or German, see^8^).

A recent step towards addressing the deficit in required empirical data is the Multilingual Eye Movement Corpus (MECO^6,9^). MECO is a coordinated international effort of eye-tracking labs and researchers dedicated to creating a database of text reading behaviour across languages. Participants in the MECO study read texts in their first language (L1) and in English as the second (or additional) language (L2) while their eye movements are recorded using an eye-tracker, an infrared video-based tracking system. Participants further complete a questionnaire collecting demographic information and language background and use, go through a brief non-verbal intelligence assessment, and complete additional tests of component skills of reading in both their L1 and L2. The MECO project makes use of highly comparable texts in different languages, participants of generally similar skill level in their L1 (university students), and similar equipment (EyeLink eye-trackers) and procedures.

Recently, the first wave of the MECO project was released, making publicly available data from 13 countries and languages. Two papers present the Wave 1 MECO data: One paper including the L1 eye-tracking reading data and relevant participant-level measures^6^ and a second paper with the L2 (i.e., English) eye-tracking reading data with associated English skill assessments^9^. Participants’ languages included in MECO’s Wave 1 represent substantial improvement in terms of linguistic diversity compared to typical studies in the field: The 13 languages in MECO’s Wave 1 span five language families (Indo-European, Koreanic, Semitic, Turkic, and Uralic) and three types of writing systems (alphabetic, e.g., English; abjad, e.g., Hebrew; and hangul, Korean). The open-source datasets made available gave a substantial boost to cross-linguistic research on oculomotor control in reading, first and second language acquisition, and computational models of text processing (for examples of studies making secondary use of the MECO Wave 1 see^10,11^).

However, it is important to keep in mind that Wave 1 of the MECO project is still highly limited in terms of cross-linguistic coverage: While the availability of data from 13 languages is a vast improvement compared to typical studies in the field (see above and Siegelman *et al*.^6^), this number still pales in comparison to the vast number of the world’s languages. The present paper therefore reports data from a new wave of the MECO project – MECO Wave 2, which includes new data from a total of 654 participants from 16 participant samples. Specifically, it makes available the eye-tracking record of reading in L1, along with the supplementary questionnaires and tests of component skills of L1 reading. Wave 2 of MECO follows the same procedures and recruitment practices as Wave 1.

Importantly, MECO’s Wave 2 features seven new languages and eight new written languages (Basque, Mandarin Chinese – with separate samples for the traditional and simplified scripts, Danish, Hindi, Icelandic, Brazilian Portuguese, and Serbian). This substantially expands the overall coverage of the MECO project to an unprecedented set of 21 languages overall. It is worth noticing that – with the exception of Chinese – all new languages added in MECO Wave 2 have had little to no coverage in the recent eye-tracking literature according to recent bibliometric analyses by Siegelman *et al*.^6^ and Angele and Duñabeitia^7^. Besides the mere increase in the number of languages, additional languages also include two new types of writing systems not represented in MECO’s Wave 1: logographic (Chinese) and abugida (Hindi). This expansion further enhances the coverage of languages and writing systems that is necessary for generalizable theories and models of reading.

Another important feature of MECO’s Wave 2 is the introduction of several “replication” samples, i.e., multiple datasets from the same language collected in different labs and, in some cases, different countries. With the new Wave 2 data, the MECO project now includes multiple samples in five languages – English, German, Hindi, Russian, and Spanish. This replication is methodologically important. Multiple within-language samples can represent regional varieties or differences in educational or social backgrounds of readers and differences in the entry requirements that different universities impose on students within a country. Replication samples can also help estimate inevitable uncontrollable factors that may lead to differences in data quality within a language, such as a given lab’s assistant training, equipment, etc. With multiple samples representing a given language, researchers can begin to dissociate behaviours characteristic of all readers of the language from behaviours characteristic of a specific university sample.

A final goal of MECO Wave 2 is to complement some of the language samples reported earlier as part of Wave 1. The data collection phase of MECO Wave 1 was interrupted by the COVID-19 pandemic and lab closures. For this reason, several sites did not reach the recommended sample size of 45–50 participants per site. The present update of the project adds data to two language samples from MECO Wave 1 (Norwegian and Turkish), bringing them up to and beyond the recommended sample size. To clarify, the supplement data from these sites are not meant to be used in isolation but rather in combination with data from MECO Wave 1^6^ from the same sites.

The present paper presents the L1 (first language) component of Wave 2 of the MECO project, describes in detail all relevant procedures and data, and establishes the data reliability, to ensure that it is appropriate for data mining by the research community. Details on how to access the publicly available MECO data are also provided.

## Methods

### Testing sites and investigated languages

Data were collected at 16 testing sites in 15 countries, representing 13 unique L1s. Table 1 presents information about the location of the testing sites included in the current release and the L1 investigated in each site. Following Siegelman *et al*.^6^, Table 1 further includes an estimate of the prevalence of each investigated language in eye-movement studies of reading, based on the bibliometric analysis of eye-movement research literature in 2000–2018. The estimated prevalence of 9 out of 13 languages included in the current MECO release was very low (<1%), suggesting that much of the new data comes from languages under-represented in research in the field. Table 2 includes information about each L1’s language family and branch, script, morphological type, and orthographic transparency (as classified in past studies^12–14^).

**Table 1:** Information about the location of testing sites, the language investigated in each site, and the prevalence of each investigated language in eye-movement studies of reading.

Sample Code	Institution	Country	Language	% of studies (2000–2018)
ba	Basque Center on Cognition, Brain and Language	Spain	Basque	<1
bp	Federal Universities of Ceara and Minas Gerais	Brazil	Brazilian Portuguese	<1
ch_s	University of Science and Technology Beijing	China	Chinese, Mandarin (simplified)	11
ch_t	National Taiwan Normal University	Taiwan	Chinese, Mandarin (traditional)	
da	Aalborg University	Denmark	Danish	<1
en_uk	University of Southampton	UK	English (UK)	57.5
ge_po	University of Potsdam	Germany	German	9.7
ge_zu	University of Zurich	Switzerland	German (Swiss)	
hi_iitk	Indian Institute of Technology Kanpur	India	Hindi	<1
hi_iiith	International Institute of Information Technology Hyderabad	India	Hindi	
ic	University of Iceland	Iceland	Icelandic	<1
no	University of Oslo	Norway	Norwegian	<1
ru_mo	Higher School of Economics	Russia	Russian	<1
se	Universities of Belgrade and Novi Sad	Serbia	Serbian	<1
sp_ch	Pontificia Universidad Católica de Valparaíso	Chile	Spanish (Chile)	4.1
tr	Middle East Technical University	Turkey	Turkish	<1

**Table 2:** Information regarding the properties of the investigated language in each site.

Sample Code	Language	Language Family (Branch)	Script (Script Type)	Morphological Typology	Orthographic Transparency
ba	Basque	Isolate	Latin (alphabetic)	Agglutinative	Transparent
bp	Brazilian Portuguese	Indo-European (Romance)	Latin (alphabetic)	Synthetic, Fusional	Transparent
ch_s	Chinese, Mandarin (simplified)	Sino-Tibetan	Chinese (logographic)	Isolating, Analytic	Opaque
ch_t	Chinese, Mandarin (traditional)	Sino-Tibetan	Chinese (logographic)	Isolating, Analytic	Opaque
da	Danish	Indo-European (North Germanic)	Latin (alphabetic)	Moderately Analytic	Opaque
en_uk	English (UK)	Indo-European (West Germanic)	Latin (alphabetic)	Moderately Analytic	Opaque
ge_po	German	Indo-European (West Germanic)	Latin (alphabetic)	Synthetic, Fusional	Moderate
ge_zu	German (Swiss)				
hi_iitk	Hindi	Indo-European (Indo-Aryan)	Devanagari (Abugida)	Synthetic, Fusional	Transparent
hi_iiith	Hindi	Indo-European (Indo-Aryan)	Devanagari (Abugida)	Synthetic, Fusional	Transparent
ic	Icelandic	Indo-European (North Germanic)	Latin (alphabetic)	Synthetic, Fusional	Moderate
no	Norwegian	Indo-European (North Germanic)	Latin (alphabetic)	Synthetic, Fusional	Moderate
ru_mo	Russian	Indo-European (East Slavic)	Cyrillic (alphabetic)	Synthetic, Fusional	Moderate
se	Serbian	Indo-European (South Slavic)	Latin (alphabetic)	Synthetic, Fusional	Transparent
sp_ch	Spanish (Chile)	Indo-European (Romance)	Latin (alphabetic)	Synthetic, Fusional	Transparent
tr	Turkish	Turkic (Oghuz)	Latin (alphabetic)	Agglutinative	Transparent

### Participants

Overall, the present Wave 2 release of MECO-L1 includes valid data from N = 654 participants (this number only includes participants that were included after data cleaning, see below). Table 3 includes information about the number of participants in each site and compensation information. Table 4 contains basic demographic information (age and years of education) along with participants’ self-rated levels of proficiency in their L1 (in speaking, oral comprehension, and reading; the demographic and self-ratings of proficiency were collected using a language background questionnaire, see *Additional Questionnaires and Tests*, below). Full demographic information for all participants is available at the project’s OSF page (see Data Records). Table 3 also provides designations for the status of each language sample in the MECO project. As noted above, eight *new* samples of languages and writing systems were added to the MECO project by nine participating sites. Five additional sites added *replication* samples for four languages, i.e., languages that were included in MECO Wave 1^6^: All these sites were different from the sites of data collection for Wave 1. Two more sites provided *supplement* samples, i.e., continued data collection initiated and reported in Wave 1 to bring their sample sizes to the expected sample size (combined with Wave 1 data, the total sample size for the Turkish and Norwegian sites is N = 45 and N = 61, respectively). Table 5 shows the main features of the current (i.e., second) Wave of MECO-L1 and how it compares to the previous Wave 1 of the project in Siegelman *et al*.^6^. To clarify, in the current release we make available new eye-movement data on L1 reading along with accompanying measures of individual differences in L1 and thus expand the scope of the MECO-L1 dataset considerably (see Data Records for details).

**Table 3:** Information regarding participants in each testing site.

Sample Code	Language	n	Sample Type	Participants’ compensation	Trials after trimming, %	Data points after trimming
ba	Basque	39	New	10 Euros/hour	65	42964
bp	Brazilian Portuguese	56	New	Volunteer	72	89457
ch_s	Chinese, Mandarin (simplified)	39	New	70 RMB/hour	88	55901
ch_t	Chinese, Mandarin (traditional)	38	New	400 NTD/session	77	51850
da	Danish	30	New	Course credit	65	37612
en_uk	English (UK)	50	Replication	Course credit	80	84364
ge_po	German	40	Replication	12.5 Euros/hour	68	54903
ge_zu	German	45	Replication	25 CHF/session	76	69589
hi_iitk	Hindi	54	New	Course credit	88	107516
hi_iiith	Hindi	57	New	200 INR/hour	85	108759
ic	Icelandic	45	New	Course credit	76	73022
no	Norwegian	19	Supplement	300 NOK gift card/session	50	20121
ru_mo	Russian	38	Replication	500 Rubles/session	67	48831
se	Serbian	43	New	Course credit	61	49561
sp_ch	Spanish (Chile)	44	Replication	Volunteer	62	67896
tr	Turkish	16	Supplement	50 Turkish Lira (~$10US)/ session	63	17224

**Table 4:** Information regarding participants’ demographics and self-rated proficiency in each testing site.

Sample Code	Mean Age (range)	Mean Years of Education (SD)	Mean Self-rating: Speaking (SD)	Mean Self-rating: Oral comp (SD)	Mean Self-rating: Reading (SD)
ba	22.41 (18–29)	15.92 (2.56)	9.17 (0.81)	9.41 (0.79)	9.38 (0.67)
bp	21.89 (18–30)	17.48 (3.24)	8.11 (1.14)	9.04 (0.93)	8.88 (0.95)
ch_s	22.38 (20–25)	16.79 (2.18)	8.59 (1.41)	8.64 (1.37)	8.44 (1.05)
ch_t	24.24 (20–30)	16.45 (2.16)	9.24 (0.97)	9.16 (0.95)	9.18 (0.83)
da	23.37 (21–35)	14.4 (1.55)	9.50 (0.68)	9.53 (0.73)	8.93 (1.05)
en_uk	19.72 (18–32)	14.08 (2.94)	9.76 (0.63)	9.78 (0.51)	9.52 (1.21)
ge_po	24.51 (18–58)	15.64 (4.1)	9.38 (0.78)	9.68 (0.53)	9.38 (0.85)
ge_zu	23.84 (18–29)	15.45 (2.56)	9.60 (0.62)	9.69 (0.56)	9.49 (0.69)
hi_iitk	21.43 (18–29)	17.39 (2.55)	8.28 (1.46)	8.72 (1.42)	7.41 (1.92)
hi_iiith	20.84 (19–23)	16.32 (2.47)	9.02 (0.99)	9.05 (0.85)	8.12 (1.4)
ic	23.42 (18–30)	15.18 (1.99)	9.32 (0.85)	9.44 (0.69)	9.31 (0.88)
no	25.16 (19–30)	16.79 (2.17)	9.37 (0.9)	9.58 (0.69)	9.47 (0.7)
ru_mo	20.85 (18–30)	13.76 (2.35)	9.32 (0.96)	9.55 (0.76)	9.58 (0.64)
se	19.53 (18–32)	12.45 (1.28)	9.75 (0.54)	9.75 (0.49)	9.7 (0.56)
sp_ch	21.30 (18–31)	15.3 (2.04)	9.23 (1.22)	9.2 (1.07)	9.25 (0.97)
tr	23.31 (20–27)	16.44 (1.63)	9.50 (0.73)	9.63 (0.81)	9.75 (0.45)

Notes: *ba:* Basque*; bp –* Brazilian Portuguese; *ch_s –* Chinese simplified*; ch_t –* Chinese traditional*; da –* Danish*; en_uk –* English (UK sample)*; ge_po -* German (Potsdam sample)*; ge_zu -* German (Zurich sample)*; hi_iiith -* Hindi (Hyderabad sample)*; hi_iitk -* Hindi (Kanpur sample)*; ic –* Icelandic*; no –* Norwegian*; ru_mo –* Russian (Moscow sample)*; se –* Serbian*; sp_ch –* Spanish (Chile sample)*; tr -* Turkish.

**Table 5:** Comparison of the first and current (i.e., second) releases of MECO-L1.

	First release: Siegelman *et al*.^6^	Current (i.e., second) release
Testing sites	13	16
*Replication samples*^*a*^	*NA*	*5*
*Supplement samples*^*b*^	*NA*	*2*
Total sample size	580	654
Written languages^*c*^	13	15
*New to MECO*	*13*	*8*
*Under-represented*^*d*^	*7*	*9*
*Use Non-Latin Script*	*4*	*4*

Notes: ^a^Replication samples are datasets from a language that already existed in an earlier MECO wave. ^b^Supplement samples provide additional data from an existing site to increase its sample size. ^*c*^The two samples of Mandarin Chinese, using the traditional and simplified script, were counted separately for this purpose. ^d^Under-represented in research means languages with a prevalence of less than 1% of eye-movement studies of reading per previous bibliometric analysis^6^.

The project obtained a general ethics approval by the McMaster University’s Ethics Review Board protocol #1892. Ethics clearance was further obtained by the following local ethics research boards: Ethics Committee at Basque Center on Cognition Brain and Language (approval number: 070521MK); Research Ethics Committee for Human Subjects at the Federal University of Ceará (CEP/UFC; approval number: 5.360.941, CAAE 56014522.6.0000.5054); Research Ethics Committee, National Taiwan Normal University (approval number: 202104HS001), University of Southampton Ethics Committee (submission number: 55085); Institutional Ethics Committee, Indian Institute of Technology Kanpur (approval number: IITK/IEC/2022-23/I/2); Institute Review Board, International Institute of Information Technology, Hyderabad (proposal number: IIITH-IRB-PRO-2024-01); the Institutional Review Board of the National Research University Higher School of Economics (HSE IRB; dated: 11/10/2020); Departmental Ethics Committee of the Department of Psychology, Faculty of Philosophy, University of Novi Sad (submission number: 202211062255_rbqs); Bioethics and Biosafety Committee of Pontificia Universidad Católica de Valparaíso (BIOEPUCV-H 335–2020); and the Human Research Ethics Committee of Middle East Technical University, Ankara, Turkey. In all other data collection sites (University of Science and Technology Beijing; Aalborg University; University of Potsdam; University of Zurich; University of Iceland; and University of Oslo), the research was declared exempt by the local ethics board given local guidelines. Participants in all sites provided informed consent for participation and for sharing of their deidentified data.

### Materials

The core data in the MECO project comes from the passage reading task, during which the participants’ eye movements are recorded. The task was identical to MECO Wave 1^6^, and its reading materials in each language were created using an identical procedure. Participants in all sites read 12 texts in their respective L1 while their eye-movement were recorded: Texts were encyclopaedic (Wikipedia-like) entries on topics such as historical figures, events, and natural or social phenomena, with topics chosen to minimize the effect of specific academic knowledge and cultural biases. As in MECO’s Wave 1, we used the 12 texts in English as our starting point. Five of the 12 texts were translated closely into the L1 of each site (these five texts are henceforth labelled as “matched texts”), through an iterative process of (human) back-translation from the target L1 to English and introduction of changes as needed. The remaining seven texts were created by the research team in each site: These texts were on the same topics as the English originals and used the same encyclopaedic genre, similar length, and a roughly similar level of difficulty (e.g., they all avoided uncommon grammatical constructions). However, they were not matched on their semantic content (we label these seven texts as “non-matched texts”). The main rationale behind including both semantically matched and non-matched texts is to enable testing which cross-linguistic similarities and differences in eye-movements are found regardless of whether texts are semantically similar or not (see^6^ for details). Each of the 12 texts was followed by four yes/no comprehension questions: Simple questions that tapped into factual knowledge obtained from the read materials and served as attention checks. The comprehension questions were similar across languages in matched texts but naturally differed for non-matched texts. Table 6 details the number of words and sentences in each text in each language.

**Table 6:** Number of words and sentences in each text in each language.

Text #	Topic		ba	bp	ch_s	ch_t	da	en	ge	hi	ic	no	ru	se	sp	tr
1*	Janus	#sent	10	10	7	7	10	10	10	10	10	10	10	10	9	10
		#word	139	188	178	180	176	182	174	214	178	177	151	167	210	146
2	Shaka	#sent	12	10	5	5	8	7	9	8	11	8	7	9	7	8
		#word	154	196	153	156	156	183	159	187	197	177	145	185	190	131
3*	Doping	#sent	9	9	8	8	9	10	9	9	9	9	9	9	9	9
		#word	162	203	188	200	175	186	190	225	159	176	155	173	237	156
4	Thylacine	#sent	11	9	6	6	11	9	11	9	9	10	9	10	7	9
		#word	140	194	178	191	172	182	180	176	195	186	190	154	169	167
5	World Environment Day	#sent	8	6	5	5	10	8	11	8	12	8	9	11	6	8
		#word	161	174	174	185	165	167	152	163	179	158	139	170	182	139
6	Monocle	#sent	12	7	4	4	11	8	10	7	8	8	8	12	11	10
		#word	125	173	144	144	164	152	149	148	175	149	129	173	212	142
7*	Wine Tasting	#sent	8	8	7	7	8	8	10	8	9	9	9	8	8	8
		#word	150	216	172	175	197	199	198	239	182	188	165	183	230	150
8	Orange Juice	#sent	9	8	5	5	10	6	9	7	9	11	7	6	8	11
		#word	111	138	144	147	154	136	132	155	182	174	138	116	160	126
9	Beekeeping	#sent	9	8	4	4	13	11	9	8	16	16	6	8	10	9
		#word	131	193	184	189	210	200	173	158	183	243	150	122	187	149
10	National Flag	#sent	12	7	6	6	10	11	11	9	16	11	11	9	10	9
		#word	151	170	172	175	178	180	180	183	183	177	149	154	234	127
11*	International Union for Conservation of Nature	#sent	7	8	6	6	8	8	8	8	7	9	8	8	8	8
		#word	143	187	168	176	165	176	171	204	168	170	164	158	225	139
12*	Vehicle Registration Plate	#sent	8	8	7	7	8	8	9	8	8	8	8	8	8	8
		#word	130	179	169	177	154	164	160	208	152	146	156	156	176	125

Notes: *ba:* Basque*; bp –* Brazilian Portuguese; *ch_s –* Chinese simplified*; ch_t –* Chinese traditional*; da –* Danish*; en_uk –* English (UK sample)*; ge_po -* German (Potsdam sample)*; ge_zu -* German (Zurich sample)*; hi_iiith -* Hindi (Hyderabad sample)*; hi_iitk -* Hindi (Kanpur sample)*; ic –* Icelandic*; no –* Norwegian*; ru_mo –* Russian (Moscow sample)*; se –* Serbian*; sp_ch –* Spanish (Chile sample)*; tr -* Turkish.

To evaluate the quality of translations for matched texts, we ran computational analyses of the meaning and textual features of the back-translations provided by different research teams (we used the back-translations, rather than the original texts written in the different L1s, because validated computational tools for textual analyses across many languages are still limited). First, to ensure that matched texts were indeed similar in their meaning across sites, we quantified the text-wise cosine semantic similarity between back-translations and the English originals using pre-trained Latent Semantic Analysis (LSA) vectors^15^. As expected, back-translations of matched texts were highly similar to the English originals (mean cosine = 0.89), significantly more than the similarity of non-matched texts to the original non-matched texts (mean cosine = 0.64, *p* < 0.001). The similarity of matched back-translated texts was on par with similarity estimates in MECO’s Wave 1^6^ (with mean cosine = 0.88) and a seminal cross-language eye-movement study that used back-translated matched texts^16^ (with mean cosine = 0.93). Second, to examine potential differences in textual content more broadly, we computed 10 complexity and readability measures for the back-translations using Coh-Metrix^17,18^. As readability measures, we included Flesch-Kincaid readability and the more psycholinguistically informed L2 readability score^19^. For text complexity, we used eight Text Easability Principal Component Scores, which quantify the contribution of linguistic characteristics to text difficulty^17^: Narrativity, simplicity, concreteness, cohesion, deep cohesion, verb cohesion, connectivity and temporality (see detailed documentation at www.cohmetrix.com). We found that the properties of matched texts were highly similar across languages: A regression analysis predicting each readability/complexity metric from “language” (a dummy-coded variable) showed no significant effect on any of the 10 complexity/readability dependent variables (see Table 7). In non-matched texts, there were some differences in readability/complexity (significant differences in 2 out of 10 readability/complexity dependent measures after Bonferroni correction). Tables 8, 9 further reports means and SDs of all readability/complexity measures by language for matched and non-matched texts, respectively, and the project’s repository includes estimates of all 10 complexity and readability measures for all texts in all languages. Future users of the MECO data can use this information both to examine how complexity/readability impacts eye-movements, and to control for these text-level properties when examining cross-linguistic effects that may be impacted by the differences found in the readability/complexity of non-matched texts.

**Table 7:** Analyses of readability and complexity metrics, for matched and non-matched texts (see^17^ for information about the text metrics).

Dependent Variable	Matched texts: F-value	Matched texts: p-value (corrected)	Unmatched texts: F-value	Unmatched texts: p-value (corrected)
Flesch-Kincaid readability	0.23	0.996 (1)	3.11	0.001 (0.012)
L2 readability	0.97	0.493 (1)	1.88	0.049 (0.495)
PC narrativity	0.21	0.997 (1)	2.63	0.005 (0.054)
PC simplicity	0.24	0.995 (1)	3.28	0.001 (0.007)
PC concreteness	0.04	0.999 (1)	0.55	0.873 (1)
PC cohesion	0.21	0.998 (1)	1.42	0.175 (1)
PC deep cohesion	0.45	0.936 (1)	1.26	0.260 (1)
PC verb cohesion	1.17	0.332 (1)	0.74	0.710 (1)
PC connectivity	0.04	0.999 (1)	0.8	0.649 (1)
PC temporality	0.21	0.997 (1)	0.88	0.569 (1)

*P*-values are reported both before and after a Bonferroni correction for multiple comparisons.

**Table 8:** Mean readability/complexity estimates by language for matched texts (SD in parentheses).

Dependent Variable	ba	bp	ch	da	en	ge	hi	ic	no	ru	se	sp	tr
Flesch-Kincaid readability	12.78 (2.81)	13.46 (2.51)	14.82 (2.13)	13.25 (2.44)	13.69 (2.52)	13.17 (2.45)	13.49 (2.39)	12.66 (2.28)	13.09 (2.61)	13.6 (2.46)	13.37 (2.55)	13.62 (2.48)	13.63 (2.58)
L2 readability	13.03 (3.27)	13.15 (3.58)	10.23 (3.31)	13.52 (3.4)	10.9 (3.40)	12.04 (2.55)	12.75 (3.62)	15.79 (4.12)	11.99 (2.99)	9.48 (2.75)	12.37 (4.36)	11.42 (4.94)	12.66 (4.05)
PC narrativity	−1.09 (0.42)	−0.94 (0.24)	−0.97 (0.42)	−0.96 (0.43)	−1.16 (0.53)	−1.06 (0.43)	−1.19 (0.44)	−0.87 (0.54)	−1.1 (0.53)	−1.10 (0.48)	−0.97 (0.62)	−0.98 (0.43)	−1.11 (0.45)
PC simplicity	−0.72 (0.49)	−0.78 (0.35)	−0.72 (0.58)	−0.75 (0.51)	−0.66 (0.45)	−0.72 (0.31)	−0.72 (0.25)	−0.61 (0.67)	−0.43 (0.29)	−0.53 (0.30)	−0.62 (0.34)	−0.7 (0.41)	−0.63 (0.47)
PC concreteness	0.6 (1.35)	0.55 (1.19)	0.68 (1.42)	0.8 (1.18)	0.8 (1.17)	0.65 (0.94)	0.46 (1.16)	0.48 (1.21)	0.64 (0.97)	0.47 (1.1)	0.6 (1.06)	0.61 (1.28)	0.60 (1.09)
PC cohesion	0.52 (0.4)	0.65 (0.93)	0.69 (1.12)	0.64 (0.99)	0.56 (0.69)	0.36 (0.72)	0.64 (0.62)	0.77 (0.93)	0.22 (0.64)	0.53 (0.38)	0.62 (0.76)	0.78 (0.81)	0.46 (0.60)
PC deep cohesion	0.61 (1.26)	0.2 (1.04)	0.48 (0.25)	0.71 (0.53)	0.36 (1.08)	0.83 (0.69)	0.58 (1.23)	0.69 (0.46)	1.25 (0.96)	0.57 (0.8)	0.61 (1.33)	0.15 (1.1)	0.31 (1.07)
PC verb cohesion	0.44 (1.02)	−0.03 (0.76)	−0.6 (0.78)	0.21 (0.48)	−0.36 (0.45)	0.56 (0.6)	0.09 (0.59)	0.4 (0.45)	0.17 (0.55)	−0.3 (0.95)	−0.12 (0.83)	−0.13 (0.51)	−0.01 (0.77)
PC connectivity	−2.65 (2.24)	−2.58 (1.71)	−2.82 (1.79)	−2.85 (1.86)	−2.74 (1.81)	−2.95 (2)	−2.68 (1.95)	−2.45 (2.39)	−2.76 (2.46)	−3.06 (1.64)	−2.57 (1.33)	−2.67 (1.83)	−2.73 (1.93)
PC temporality	−0.26 (1.36)	−0.48 (1.31)	0.04 (2.02)	−0.59 (1.05)	−0.59 (1.99)	−0.3 (1.31)	−0.71 (1.13)	−0.07 (0.93)	−0.28 (1.81)	0.1 (1.22)	−0.65 (1.33)	−0.78 (1.76)	−0.04 (1.13)

Notes: *ba:* Basque*; bp –* Brazilian Portuguese; *ch_s –* Chinese simplified*; ch_t –* Chinese traditional*; da –* Danish*; en_uk –* English (UK sample)*; ge_po -* German (Potsdam sample)*; ge_zu -* German (Zurich sample)*; hi_iiith -* Hindi (Hyderabad sample)*; hi_iitk -* Hindi (Kanpur sample)*; ic –* Icelandic*; no –* Norwegian*; ru_mo –* Russian (Moscow sample)*; se –* Serbian*; sp_ch –* Spanish (Chile sample)*; tr -* Turkish.

**Table 9:** Mean readability/complexity estimates by language for non-matched texts (SD in parentheses).

Dependent Variable	ba	bp	ch	da	en	ge	hi	ic	no	ru	se	sp	tr
Flesch-Kincaid readability	10.13 (2.38)	12.76 (2.23)	14.11 (2.23)	10.59 (1.17)	12.9 (1.79)	10.78 (1.29)	10.68 (1.38)	10.57 (1.95)	10.98 (1.44)	12.22 (1.58)	11.33 (1.17)	13.28 (3.18)	12.06 (1.44)
L2 readability	14.71 (4.79)	11.52 (3.63)	13.45 (1.97)	16.38 (6.07)	9.7 (6.88)	14.68 (4.51)	17.15 (4.75)	16.67 (6.05)	14.36 (4.86)	11.6 (4.36)	13.1 (2.11)	10.59 (4.58)	10.95 (4.45)
PC narrativity	−0.76 (0.19)	−1.31 (0.27)	−0.81 (0.58)	−1.04 (0.31)	−1.17 (0.25)	−1.2 (0.27)	−0.79 (0.22)	−0.52 (0.55)	−1.21 (0.17)	−0.8 (0.45)	−1.14 (0.46)	−0.98 (0.42)	−0.84 (0.46)
PC simplicity	0 (0.34)	−0.68 (0.64)	−1.16 (0.39)	−0.08 (0.3)	−0.55 (0.44)	−0.03 (0.57)	−0.19 (0.4)	−0.67 (0.46)	−0.26 (0.49)	−0.74 (0.35)	−0.01 (0.78)	−0.82 (0.93)	−0.67 (0.69)
PC concreteness	1.01 (0.74)	1.15 (0.28)	1.33 (0.73)	1.08 (0.74)	1.14 (0.97)	0.99 (1.14)	1.25 (0.73)	1.01 (0.46)	1.7 (0.73)	1.03 (0.83)	1.16 (0.57)	0.97 (0.83)	0.86 (0.69)
PC cohesion	−0.33 (0.5)	0.09 (0.78)	0.95 (1.08)	0.54 (0.72)	0.33 (0.98)	0.1 (1.6)	0.87 (0.71)	1.04 (0.9)	0.77 (0.74)	0.49 (1.02)	0.18 (0.81)	−0.04 (1.06)	0.23 (0.63)
PC deep cohesion	−0.36 (1.23)	0.23 (0.96)	0.2 (1.5)	0.12 (1.14)	1.25 (1.31)	1.1 (1.85)	0.66 (0.54)	0.03 (1.2)	1.03 (1.29)	1.12 (1.16)	0.77 (1.1)	0.99 (0.78)	1.08 (1.5)
PC verb cohesion	0.17 (0.97)	0.06 (0.97)	−0.56 (1.44)	0.57 (0.91)	−0.33 (0.93)	0.72 (1.72)	−0.16 (0.86)	0.55 (1.79)	0.09 (0.88)	−0.27 (1.34)	0.43 (1.84)	0.08 (0.51)	−0.24 (0.63)
PC connectivity	−3.07 (1.83)	−2.3 (2.59)	−2.44 (1.6)	−2.98 (2.06)	−2.54 (1.02)	−3.21 (1.04)	−3.02 (1.23)	−3.5 (1.44)	−3.95 (1.92)	−2.53 (1.81)	−3.49 (1.82)	−3.1 (1.31)	−1.94 (1.02)
PC temporality	−0.73 (1.09)	−1.47 (1.61)	−0.68 (1.55)	−0.51 (1.24)	−0.58 (1.04)	0.22 (1.3)	−0.29 (1.06)	−0.61 (0.95)	−1.11 (1.34)	−0.64 (0.92)	−0.88 (1.65)	0.16 (1.32)	−0.3 (1.54)

Notes: *ba:* Basque*; bp –* Brazilian Portuguese; *ch_s –* Chinese simplified*; ch_t –* Chinese traditional*; da –* Danish*; en_uk –* English (UK sample)*; ge_po -* German (Potsdam sample)*; ge_zu -* German (Zurich sample)*; hi_iiith -* Hindi (Hyderabad sample)*; hi_iitk -* Hindi (Kanpur sample)*; ic –* Icelandic*; no –* Norwegian*; ru_mo –* Russian (Moscow sample)*; se –* Serbian*; sp_ch –* Spanish (Chile sample)*; tr -* Turkish.

### Additional questionnaires and tests

In addition to the main passage reading eye-tracking task, participants in all sites completed two identical instruments: (1) The non-verbal IQ test from the Culture Fair Test-3 (CFT20, Subset 3 Matrices, short version, Form A, timed at 3 minutes^20^), and (2) an abridged version of the Language Experience and Proficiency Questionnaire (LEAP-Q^21^). The CFT20 aimed at providing a comparable measure of non-verbal intelligence across all sites (due to copyright restrictions, however, it was not available in 2 out of 16 sites: Brazil and Serbia). The LEAP-Q aimed at collecting basic demographic and linguistic information about participants. These two instruments were identical to MECO’s Wave 1^6^. Researchers at various sites were also encouraged to include additional (non-eye-tracking) measures of individual differences in L1 reading and language proficiency, with the goal of enabling within-site analyses of the correlations between individual differences in language skills and oculomotor reading behaviour. Note that these measures were not shared across sites, given differences in the availability of measures in different languages. Common L1 individual differences tests included measures of vocabulary, word and pseudoword naming, phonological awareness, and other component skills of reading. The full individual-differences data from each site are available at the project’s OSF page (see Data Records).

### Procedure

The order of task administration was fixed in all sites: Participants started by filling out the LEAP-Q questionnaire, followed by the main reading task in their first language during which their eye movements were recorded, and then the individual-differences battery (including the CFT-20 and any L1 individual-differences tests). The entire procedure lasted no more than an hour, and breaks were provided as needed. At the conclusion of the experimental session, participants in all samples proceeded to participate in an English-language eye-tracking study (the “MECO-L2” component of the project). The goal of that study was to create an additional eye-tracking corpus of reading in English as a non-dominant language. This additional study is beyond the scope of this paper and is reported elsewhere^22^. However, participant identifiers are shared between the data releases of MECO’s components, to enable within-participant analyses of reading patterns in L1 and L2 (see^9^).

### Eye-tracking task: apparatus and procedure

To register eye-movements during the reading task, all sites used an EyeLink eye-tracker (SR Research, Kanata, Ontario, Canada). The exact model in each lab varied (with labs using either the Portable Duo, EyeLink II, 1000 or 1000+ models). Sampling rate was set at 1000 Hz, with the exception of one lab which used the EyeLink II model with the sampling rate of 500 Hz. All sites used the same experimental task template programmed in the Experiment Builder software (SR Research). A chin and a forehead rest were used to minimize head movements. Before the beginning of the task, a 9-point calibration was performed (i.e., with nine targets distributed around the display), followed by a 9-point accuracy test for validation. Experimenters were also encouraged to perform re-calibrations whenever deemed necessary during the reading task. Stimuli were viewed binocularly but eye-movements were analysed from the self-reported dominant eye only. Before each trial (i.e., passage), a drift correction was performed, via a dot appearing slightly to the left of the first word in the passage. Once the participant had fixated on it, the trial began. Calibration was monitored by the experimenter throughout the task and was redone if necessary. Each of the 12 passages appeared on a separate screen, with participants instructed to read them silently for comprehension and press the space bar when finished. Each text was then followed by the four yes/no comprehension questions, appearing one after the other on separate screens, with participants instructed to provide their answer using the 1 (“yes”) and 0 (“no”) keys –the comprehension accuracy data are made available as part of the data release. Texts were presented in a mono-spaced font, except in Serbia (due to technical issues and experimenter error) and in India (because mono-spaced fonts are unavailable for Devanagari script). Different sites used font settings to maximize the readability of texts given their local setup (i.e., screen size and resolution). Table 10 summarizes the specifications of the apparatus and presentation settings in each participating site. The project’s OSF site further includes image files with the presented texts from all sites (i.e., bitmap images as used by the experiment presentation software).

**Table 10:** Information about apparatus and presentation settings in each site.

Sample Code	Eye-tracker	Font type	Font point size	Line spacing	Eye-to-screen distance, in cm	Characters per visual angle	Screen type	Screen resolution	Screen size, in inch
ba	Eyelink 1000	Consolas	20	1.5	60	2.5	Viewsonic CRT	1600 × 1024	19
bp	EyeLink 1000	Consolas	20	2	75	3.5	IPS-LCD	1920 × 1080	19
ch_s	Eyelink-1000+	PMingLiu	15	1.5	60	1.4	DELL P1917S	1024 × 768	19
ch_t	EyeLink 1000	PMingLiu	11	1.5	60	1.4	Dell P2417	1920 × 1080	24
da	EyeLink Portable Duo	Consolas	20	1.5	95	4.0	Dell S2421HGF	1920 × 1080	24
en_uk	Eyelink 1000	Consolas	20	2	70	2.9	ASUS VG248QE	1920 × 1080	24
ge_po	EyeLink Portable Duo	Consolas	20	1.5	91	3.9	ASUS ROG Strix XG259QN	1920 × 1080	24
ge_zu	EyeLink Portable Duo	Consolas	10	1.5	60	3.5	ASUS OMEN	1280 × 1024	24
hi_iitk	EyeLink 1000+	Kokila	28	1	70	2.2	BenQ-XL2430T	1920 × 1080	24
hi_iiith	EyeLink 1000+	Kokila	28	1	90	2.9	Dell S2415Hb	1920 × 1080	24
ic	EyeLink 1000+	Consolas	20	1.5	96	4.0	BenQ, XL2411Z	1920 × 1080	24
no	EyeLink 1000+	Consolas	20	1.5	89	3.7	BenQ XL4230-B	1920 × 1080	24
ru_mo	EyeLink 1000+	Consolas	22	1.5	90	3.4	ASUS VG248QE	1920 × 1080	24
se	EyeLink II	Tahoma	20	2	90	4.0	ViewSonic Graphics Display G90FB	1280 × 1024	19
sp_ch	EyeLink Portable Duo	Consolas	20	1.5	70	2.9	Gamer Rog Zephyrus M16	1920 × 1080	16
tr	Eye Link 1000+	Consolas	20	1.5	60	2.5	HP Pavilion 23cw	1920 × 1080	23

### Data processing and cleaning

As in all other components of the MECO project, the popEye software^23^ (implemented in R, version 0.8.3) was used to pre-process the eye-tracking data. During this pre-processing process, fixations are automatically corrected on the vertical axis and assigned to lines. In the current Wave 2 of MECO, the “slice” algorithm was used, because it was shown to substantially improve assignment accuracy compared to the baseline algorithm used for Wave 1^24^. However, in the two supplement samples (i.e., in Turkey and Norway) the “chain” algorithm was used to maintain consistency across MECO waves within a site. Following the automatic fixation alignment procedure by popEye, members of the research team inspected the output of the software and assessed the quality of the resulting data. Further, when processing data in simplified and traditional Chinese, members of the research team used the “interactive” mode of popEye to correct cases of misalignment, when possible, because inspection of the software’s output revealed cases that could be easily fixed by that mode. Texts in which fixations and text lines were misaligned after processing (e.g., due to poor calibration or software error) were removed from the data pool. Additionally, as in other MECO components, participants with fewer than 5 (out of 12) usable texts were removed altogether from the database (see Table 3 above for percent of remaining texts and word tokens after data cleaning).

## Data Records

As with previous releases of MECO^6,9,22^, the data of the current Wave 2 release of MECO L1 is made fully available via the Open Science Foundation (OSF) website^25^, at: https://osf.io/3527a/.

This OSF repository includes word-level reports from usable participants and trials, as well as passage- and sentence-level reports. Two of the scripts in the present MECO release are written without spaces: simplified and traditional Chinese. We identified words in these scripts based on the segmentation provided by linguist experts in Mandarin. We also make available fixation and saccade reports, information about participants’ reading rate (at the passage- and subject-level), and comprehension accuracy (at the word-, text- and subject-level). Note that variable names in these reports are identical and thus backward compatible with variables in other releases of the MECO project^6,9,22^. The participant identifiers in the current release are compatible with those used in the MECO L2 Wave 2 release^22^, to enable within-participant analyses of L1-L2 eye-movement data. Also included on OSF are full data from individual differences tests in L2, the non-verbal IQ test, and the background questionnaire. The OSF page also includes auxiliary data tables (e.g., detailed descriptive statistics for different eye-movement measures by sample), and the analysis code for the validation analyses that follow. Please refer to the readme files in the OSF repository for detailed information regarding files and data structures.

To clarify, the new data being released as part of the current paper is the data on L1 reading in the 16 Wave 2 testing sites, along with the accompanying individual differences data. The data that is unique to the current release includes therefore all files on the project’s OSF page under *release 2.0/version 2.0/wave 2*. As noted above, these new data include word- sentence- and passage-reports from the eye-movement record (on OSF, under: release 2.0/version 2.0/wave 2/primary data/eye tracking data) and full accompanying individual differences data (under: release 2.0/version 2.0/wave 2/primary data/individual differences data). The same OSF project includes also parallel Wave 1 data from MECO L1, reported in Siegelman *et al*.^6^ (under: release 2.0/version 2.0/wave 1). New data versions, within release 2.0, will be made available as needed (e.g., to reflect improvements in data processing pipelines). A separate OSF project includes data from MECO L2, that is, eye-movement English-as-L2 reading data and separate tests of individual differences (https://osf.io/q9h43/). This latter OSF project includes both the Wave 1 MECO L2 data (reported in^9^), and the Wave 2 MECO L2 data (reported in^22^), which can be merged with the respective Wave of MECO L1 data, given the shared participant identifiers.

## Technical Validation

As means of validation, we used two sets of analyses, also used in earlier MECO releases^6,9^. The first examines the *reliability* of the resulting data, via estimates of the stability of basic measures commonly used in eye-movement research, computed both at the item- and participant-level. This analysis is meant to ensure that the data have reasonable levels of measurement error that allow for secondary data usage and hypothesis testing. The second analysis provides basic descriptive information for eye-movement measures and accompanying measures such as comprehension accuracy.

In validation analyses, we focus on several basic eye-movement variables that are considered as fundamental measures of reading fluency. Word-level variables include skipping (a binary index of whether the word was not fixated even once during the entire text reading, labeled as *skip*); and, for words that were fixated at least once: first fixation duration (the duration of the first fixation landing on the word, *firstfix.dur*); gaze duration (the summed duration of fixations on the word in the first pass, i.e., before the gaze leaves it for the first time, *firstrun.dur*); total fixation duration (the summed duration of all fixations on the word, *dur*); number of fixations on the word (*nfix*); refixation (a binary index of whether a word elicited more than one fixation in the first pass, *refix*); regression-in (a binary index of whether the gaze returned to the word after inspecting further textual material; *reg.in*); and re-reading (a binary index of whether the word elicited fixations after the first pass, i.e., after the gaze left the word for the first time, *reread*). A detailed discussion of these variables is provided in previous studies^5,26,27^. At the text- and participant-level, we further define the following measures: reading rate (in words per minute, *rate*), and comprehension accuracy (the percent of correct responses in comprehension questions) computed for all passages (*acc*) and for matched texts only (*acc_matched*). Prior to analyses in this section, we further cleaned the data by removing data points that showed unrealistically short (<80 msec) first fixations, which are unlikely to provide sufficient time to complete visual uptake^28^, or very long total fixation times (top 1% of the participant-specific distribution, all exceeding 3 s on the word).

### Reliability estimates

We computed two types of reliability estimates: At the participant-level and at the word token-level (see also^6,9^). Both were estimated mainly using a split-half procedure. Participant-level reliability for a given dependent variable examines how stable that measure is given inter-participant variation. Using a split-half procedure, it is computed as the correlation between mean values for ‘odd’ and ‘even’ words within a participant (e.g., computing, say, mean gaze duration for words tokens 1, 3, 5, etc. and words 2, 4, 6, etc. for each participant, and examining the correlation between the two sets of values). This split-half procedure was used for all dependent variables, with the exception of reading rate where it was estimated using an Intra-class Correlation Coefficient (ICC), measuring the degree of agreement in reading rate across the 12 texts. Word-level reliability was done at the word token-level, and is of interest mainly for studies of the effect that word properties have on eye movements. For each word token in the database, mean values were computed for each eye-movement measure for “odd” and “even” participants separately. Then, the correlation across word tokens between these two sets of values form a reliability estimate at the word token-level.

Tables 11, 12 provide reliability estimates for the different dependent variables in each site, at the participant- and word token-level, respectively. As can be seen, reliability at the participant level was very high (*r’s > *0.9 in all sites for all measures after Spearman-Brown correction for attenuation). This is in line with parallel previous estimates in the MECO project^6,9,22^, and elsewhere^29^ and is expected given the general stability of basic eye-movement measures at the individual level and the large number of words read by each participant. We also computed participant-level reliability estimates for comprehension accuracy (both for all texts and matched texts only, computed across languages). In line with Siegelman *et al*., 2022, reliability for comprehension accuracy were generally lower (*r = *0.53 and *r = *0.50 for all texts and matched texts, respectively). This is expected: MECO-L1 comprehension questions are meant to serve as attention checks, not as sensitive measures of individual differences^6^. Future users should be mindful when using these metrics in correlational analyses.

**Table 11:** Reliability at the participant-level.

Language (site)	*firstfix.dur*	*firstrun.dur*	*dur*	*nfix*	*reg.in*	*reread*	*skip*	*mean r*	*rate*
Basque	0.98, 0.99	0.98, 0.99	0.99, 1.00	0.98, 0.99	0.95, 0.97	0.98, 0.99	0.88, 0.94	0.97, 0.99	0.97
Brazilian Portuguese	0.98, 0.99	0.98, 0.99	0.98, 0.99	0.98, 0.99	0.96, 0.98	0.97, 0.98	0.95, 0.97	0.97, 0.99	0.97
Mandarin, simplified	0.98, 0.99	0.98, 0.99	0.98, 0.99	0.98, 0.99	0.97, 0.98	0.98, 0.99	0.92, 0.96	0.97, 0.99	0.96
Mandarin, traditional	0.95, 0.97	0.95, 0.97	0.95, 0.97	0.94, 0.97	0.95, 0.98	0.92, 0.96	0.98, 0.99	0.95, 0.98	0.98
Danish	0.95, 0.98	0.92, 0.96	0.95, 0.97	0.97, 0.98	0.94, 0.97	0.97, 0.99	0.86, 0.92	0.94, 0.97	0.97
English (UK)	0.98, 0.99	0.99, 0.99	0.99, 1.00	0.99, 0.99	0.97, 0.98	0.98, 0.99	0.93, 0.97	0.98, 0.99	0.98
German (Potsdam)	0.98, 0.99	0.97, 0.98	0.98, 0.99	0.98, 0.99	0.95, 0.97	0.98, 0.99	0.96, 0.98	0.97, 0.99	0.99
German (Zurich)	0.99, 0.99	0.99, 0.99	0.99, 0.99	0.98, 0.99	0.97, 0.99	0.97, 0.99	0.98, 0.99	0.98, 0.99	0.99
Hindi (IIITH)	0.99, 1.00	1.00, 1.00	0.99, 1.00	0.99, 1.00	0.96, 0.98	0.99, 1.00	0.98, 0.99	0.99, 1.00	0.96
Hindi (IITK)	0.99, 0.99	0.99, 0.99	0.99, 0.99	0.99, 0.99	0.96, 0.98	0.98, 0.99	0.95, 0.98	0.98, 0.99	0.98
Icelandic	0.99, 0.99	0.99, 0.99	0.99, 0.99	0.99, 0.99	0.95, 0.97	0.98, 0.99	0.97, 0.99	0.98, 0.99	0.94
Norwegian	0.98, 0.99	0.99, 0.99	0.99, 1.00	0.98, 0.99	0.95, 0.98	0.93, 0.96	0.84, 0.91	0.97, 0.98	0.98
Russian	0.99, 0.99	0.98, 0.99	0.99, 0.99	0.98, 0.99	0.93, 0.97	0.97, 0.99	0.94, 0.97	0.97, 0.99	0.96
Serbian	0.95, 0.97	0.95, 0.98	0.96, 0.98	0.97, 0.98	0.93, 0.97	0.96, 0.98	0.85, 0.92	0.95, 0.97	0.96
Spanish (Chile)	0.97, 0.98	0.98, 0.99	0.99, 1.00	0.98, 0.99	0.95, 0.97	0.98, 0.99	0.91, 0.96	0.97, 0.99	0.95
Turkish	0.99, 1.00	0.99, 1.00	0.99, 1.00	0.99, 1.00	0.95, 0.97	0.99, 1.00	0.9, 0.95	0.98, 0.99	0.97

Values before and after the comma presents estimates before and after Spearman-Brown correction for attenuation. All reliability estimates are based on split-half procedure, except for reading rate which is based on ICC. Mean correlations are based on mean *z* values after Fisher *r*-to-*z* transformation, which were then transformed back to *r* values using an inverse transformation.

Notes: firstfix.dur: first fixation duration; firstrun.dur: gaze duration; dur: total fixation time; nfix: number of fixations; reg.in: regression rate; reread: likelihood of second pass; skip: skipping rate; mean r: mean reliability across eye-tracking measures (excluding reading rate); *rate: reading rate*.

**Table 12:** Reliability at the word token-level.

Sample L1	*firstfix.dur*	*firstrun.dur*	*dur*	*nfix*	*reg.in*	*reread*	*skip*	*mean r*
Basque	0.39, 0.57	0.77, 0.87	0.80, 0.89	0.80, 0.89	0.63, 0.78	0.49, 0.66	0.79, 0.88	0.69, 0.81
Brazilian Portuguese	0.31, 0.48	0.58, 0.73	0.74, 0.85	0.76, 0.87	0.66, 0.80	0.55, 0.71	0.91, 0.95	0.69, 0.81
Mandarin, simplified	0.23, 0.37	0.33, 0.50	0.44, 0.61	0.40, 0.58	0.46, 0.63	0.30, 0.46	0.70, 0.83	0.42, 0.59
Mandarin, traditional	0.17, 0.29	0.22, 0.36	0.42, 0.59	0.44, 0.62	0.45, 0.62	0.27, 0.42	0.56, 0.72	0.37, 0.53
Danish	0.39, 0.56	0.69, 0.82	0.73, 0.84	0.70, 0.82	0.54, 0.70	0.42, 0.59	0.81, 0.9	0.63, 0.77
English (UK)	0.42, 0.59	0.62, 0.77	0.71, 0.83	0.72, 0.83	0.71, 0.83	0.51, 0.68	0.87, 0.93	0.68, 0.80
German (Potsdam)	0.37, 0.54	0.73, 0.84	0.76, 0.87	0.77, 0.87	0.67, 0.80	0.43, 0.60	0.80, 0.89	0.67, 0.80
German (Zurich)	0.36, 0.53	0.77, 0.87	0.79, 0.88	0.80, 0.89	0.63, 0.78	0.46, 0.63	0.82, 0.90	0.69, 0.81
Hindi (IIITH)	0.33, 0.49	0.75, 0.86	0.85, 0.92	0.85, 0.92	0.61, 0.76	0.57, 0.73	0.84, 0.91	0.72, 0.84
Hindi (IITK)	0.37, 0.54	0.66, 0.80	0.83, 0.91	0.83, 0.91	0.66, 0.79	0.65, 0.79	0.85, 0.92	0.72, 0.84
Icelandic	0.40, 0.57	0.75, 0.86	0.78, 0.88	0.78, 0.87	0.66, 0.79	0.49, 0.66	0.87, 0.93	0.70, 0.82
Norwegian	0.16, 0.27	0.44, 0.61	0.50, 0.66	0.47, 0.64	0.34, 0.51	0.2, 0.33	0.57, 0.73	0.39, 0.56
Russian	0.30, 0.46	0.47, 0.64	0.53, 0.69	0.57, 0.73	0.49, 0.66	0.35, 0.52	0.89, 0.94	0.56, 0.71
Serbian	0.32, 0.48	0.62, 0.76	0.67, 0.81	0.68, 0.81	0.52, 0.68	0.41, 0.58	0.88, 0.94	0.62, 0.76
Spanish (Chile)	0.25, 0.40	0.52, 0.68	0.61, 0.76	0.65, 0.79	0.53, 0.69	0.41, 0.58	0.84, 0.91	0.57, 0.72
Turkish	0.24, 0.38	0.42, 0.59	0.45, 0.62	0.45, 0.62	0.46, 0.63	0.26, 0.41	0.67, 0.80	0.43, 0.60

Values before and after the comma presents estimates before and after Spearman-Brown correction for attenuation. All reliability estimates are based on split-half procedure. Mean correlations are based on mean *z* values after Fisher *r*-to-*z* transformation, which were then transformed back to *r* values using an inverse transformation.

Notes: firstfix.dur: first fixation duration; firstrun.dur: gaze duration; dur: total fixation time; nfix: number of fixations; reg.in: regression rate; reread: likelihood of second pass; skip: skipping rate; mean r: mean reliability across eye-tracking measures (excluding reading rate); *rate: reading rate*.

Reliability at the word token-level was somewhat lower than parallel estimates at the participant-level, again in line with similar previous estimates^6,9,30^. In particular, there were inevitably lower reliability estimates for sites with a smaller number of participants (e.g., Supplement samples in Norwegian and Turkish), and for measures that were previously shown to be less stable at the word-level (e.g., re-reading, first fixation duration^6,9,22^). Still, reliability levels found in the Wave 2 data were high on average across sites and measures (mean *r = *0.71, median 0.73; values after Spearman-Brown correction), and again comparable to those in MECO’s Wave 1^6^ as well as the GECO database^30^.

### Descriptive statistics

As another validation of the new data, we examined the inter-relations among the basic eye-movement measures, as well as their correlation with the measure of non-verbal intelligence (CFT). As shown in Table 13, the resulting patterns of correlation between these variables is highly consistent with similar previous estimates^6,9,30^. Specifically, we observe (1) substantial correlations between the various eye-movement measures; and (2) conversely, low correlations between CFT scores and eye-movement measures (|r| ≤ 0.15). These expected correlational patterns and their similarity with the MECO’s Wave 1 data further validate the new data.

**Table 13:** Correlation table for reading measures (and non-verbal intelligence) across languages (N = 654).

	(1)	(2)	(3)	(4)	(5)	(6)	(7)	(8)	(9)	(10)
1) skipping		−0.29	−0.52	−0.52	0.82	−0.58	−0.79	0.02	−0.30	0.05
2) first fixation duration	<0.001		0.90	0.79	−0.56	0.29	0.29	−0.03	0.15	0.06
3) gaze duration	<0.001	<0.001		0.86	−0.68	0.48	0.63	−0.05	0.17	0.02
4) total fixation duration	<0.001	<0.001	<0.001		−0.79	0.81	0.59	0.34	0.63	0.07
5) reading rate	<0.001	<0.001	<0.001	<0.001		−0.77	−0.73	−0.25	−0.58	0.00
6) number of fixations	<0.001	<0.001	<0.001	<0.001	<0.001		0.67	0.55	0.87	0.05
7) refixation	<0.001	<0.001	<0.001	<0.001	<0.001	<0.001		−0.03	0.26	−0.09
8) regression in	0.640	0.498	0.200	<0.001	<0.001	<0.001	0.427		0.75	0.15
9) rereading	<0.001	<0.001	<0.001	<0.001	<0.001	<0.001	<0.001	<0.001		0.12
10) cft	0.221	0.136	0.705	0.082	0.969	0.217	0.033	<0.001	0.003	

Values above the diagonal show Pearson correlations; values below the diagonal show *p* values.

**Fig. 1:**
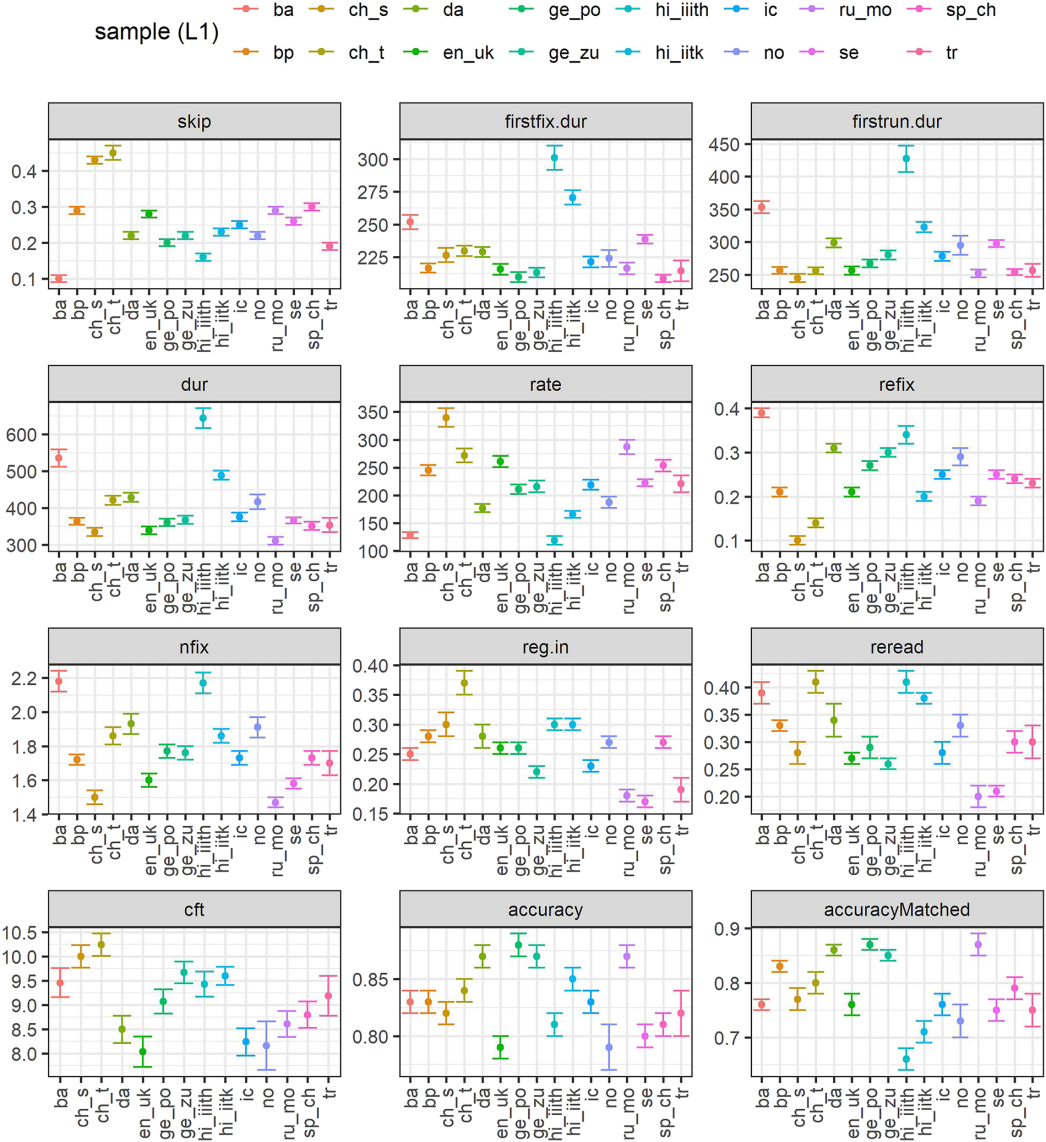
Means of eye-movement measures, comprehension accuracy, and CFT scores, across testing sites. Error bars stand for ± 1 SE. *skip*: skipping rate; *firstfix.dur*: first fixation duration (in msec); *firstrun.dur*: gaze duration (in msec); *dur*: total fixation time (in msec); *nfix*: number of fixations; *rate*: reading rate (words per minute); *refix*: likelihood of second fixation on the word; *reg.in*: regression rate; *reread*: likelihood of second pass; *accuracy*: comprehension accuracy; *accuracyMatched*: percent answers correct in matched texts; *cft*: score in the CFT test. *ba:* Basque*; bp –* Brazilian Portuguese; *ch_s –* Chinese simplified*; ch_t –* Chinese traditional*; da –* Danish*; en_uk –* English (UK sample)*; ge_po -* German (Potsdam sample)*; ge_zu -* German (Zurich sample)*; hi_iiith -* Hindi (Hyderabad sample)*; hi_iitk -* Hindi (Kanpur sample)*; ic –* Icelandic*; no –* Norwegian*; ru_mo –* Russian (Moscow sample)*; se –* Serbian*; sp_ch –* Spanish (Chile sample)*; tr -* Turkish.

Lastly, we calculated for each site the means and standard errors for all eye-movement measures and comprehension accuracy (in matched and all texts). These estimates were calculated over respective average values computed per participant. The descriptive by-site statistics, shown in Fig. 1, can be used for the purposes of both validation and comparison. In terms of validation, the ranges of means across sites resemble those in MECO’s Wave 1. For example, comprehension accuracy for matched texts in the present data ranges between 0.7 and 0.9 in 15/16 sites, a range that included mean comprehension accuracy in 12/13 sites in MECO’s Wave 1. The same is true for cumulative measures of eye-movements (e.g., first fixation duration generally ranges from 200 to 250 msec; gaze durations from 250 to 300; reading rate from 150 to 350 words/minute: All ranges are comparable to the ones observed in the samples in MECO’s Wave 1^6^). At the same time, there are clearly noticeable cross-linguistic and inter-site differences in the data. For instance, some language samples show longer fixations and larger number of fixations per word on average: see in particular patterns observed among readers of Basque (an agglutinative language) and Hindi (a morphologically rich language with a visually complex abugida writing system). Furthermore, readers of simplified and traditional Chinese demonstrate higher skipping and regression rates than readers of other languages: This is likely due to the extremely small number of (visually complex) characters that make words in written Chinese. Further variation across sites is found in essentially all dependent variables of eye-movement behaviour. Such variation is both expected and desirable. Properties of the language, the writing system, and participants in each site are expected to contribute to variation in oculomotor behaviour. Future research can therefore mine the new data to examine what constitutes systematic patterns of cross-linguistic differences and similarities and reveal the sources behind them.

### Limitations

MECO is an international collaboration that uses existing setups in different sites worldwide to collect cross-linguistic data on eye-movements during reading. The use of existing setups led to inevitable variability in parameters related to data acquisition, including the physical size of the texts as read by the participants, as determined by cross-site variation in screen size, resolution, and participants’ distance from screen (see information regarding presentation parameters in each site, and in particular the number of characters per visual angle, in Table 10). Analyses that require stricter control of these parameters may require more targeted experimental manipulations; however, for the many effects that are presumably not contingent on the physical size of the orthographic characters, this variance may be either less relevant or even an opportunity to examine whether cross-linguistic differences are present even when controlling for these differences. Similarly, different MECO sites use available student populations from their local university pool, which leads to a general reliance of the project on educated populations, and sometimes further leads to cross-site variation in participants’ demographics and educational background. We do make available information about demographics and language and educational background, which can be considered in future analyses, but we admit that in some cases a stricter control is again needed. Another limitation of MECO is the variation of sample size across sites. In this second wave of data collection we have attempted to bring the sample size in each site to a minimum of 45 participants: Indeed, across the two data collection waves, 18 out of 27 sites now reach this number (also with the help of the current supplement samples), and another 7 sites have a sample size between N = 38 and N = 44 participants. Still, the variation in sample sizes should be considered and data from sites with smaller samples should be interpreted with caution.

More broadly, we wish to highlight that analyses on MECO can and should be supplemented with both targeted manipulations and with analyses of data from language-specific eye-movement corpora, which have recently become increasingly common^31–33^. We can envision multiple scenarios where MECO can first provide insights about cross-linguistic trends, with data from language-specific corpora or experimentation then used to validate the findings and dig deeper into the observed pattern within a language or a set of languages. We also highlight that the MECO project is continuously involving to include more sites and languages and to increase the sample sizes of existing data samples via additional supplement samples. As the MECO network continues to grow, we hope to converge on a dataset that further achieves cross-linguistic coverage as well as a high statistical power for both between- and within-site analyses.

## Data Availability

The code used for the validation analyses is available via the project’s OSF repository – see Data Records.
